# Research on the protection and restoration technology of glazed components in ancient architecture

**DOI:** 10.1371/journal.pone.0352638

**Published:** 2026-07-10

**Authors:** Yao Chen, Liwen Yu, Liping Chen, Linzi Ouyang, Ning Wang

**Affiliations:** 1 School of Design Art and Architecture, Zhejiang Wanli University, Ningbo, China; 2 School of Arts and Design, Guangzhou University, Guangzhou, China; 3 School of Architecture and Design, Xinyu University, Xinyu, China; Politecnico di Milano, ITALY

## Abstract

In traditional Chinese architecture, glazed tile components are a significant decorative element that are frequently utilized on walls and roofs. However, glazed tile components typically show variable degrees of deterioration and damage owing to long-term exposure to the natural environment. This study examines and evaluates the preservation quality and primary forms of deterioration of historic architectural glazed tile components. The damage development process of glazed tile components under the combined influences of temperature, humidity fluctuations, and acidic environments was discovered through characterization of material morphological changes and corrosion products. Tetraethyl orthosilicate (TEOS) and hydroxyl-terminated polydimethylsiloxane (PDMS−OH) were chosen as reinforcement materials for common problems including pulverization of the glazed tile body. The glazed tile body was reinforced using ethanol as a solvent. The best material formulation for glazed tile body reinforcement was chosen by evaluating the performance of reinforcement systems with various ratios using a thorough index analysis. Concurrently, scanning electron microscopy (SEM) and X-ray photoelectron spectroscopy (XPS) were used to examine the reinforcing process. This work suggests modifying the matrix material’s particle size to improve mechanical characteristics and structural stability for glazed tile components that are extensively damaged and challenging to repair with reinforcement. The study’s findings can serve as a theoretical foundation and technical guide for the scientific preservation and repair of glazed tile elements found in historic buildings.

## 1. Introduction

China’s vast history and culture are preserved in its ancient architecture, which is a significant cultural legacy that highlights the political, artistic, cultural, and economic advancements of my nation’s many historical eras. These historic structures, which are primarily preserved in the outdoors, have sustained varied degrees of damage over time as a result of both human and natural causes, necessitating their immediate restoration and preservation. People have progressively realized that the study and preservation of ancient architectural heritage is a significant responsibility that calls for attention and ongoing improvement in recent years due to the state’s increased emphasis on cultural relic protection [1]. In traditional Chinese architecture, glazed architectural elements—particularly those found in roofs, walls, and ornamental structures—are significant decorative and protective features. These materials frequently undergo progressive deterioration, including cracking, pulverization, and surface corrosion, as a result of prolonged exposure to environmental elements such temperature swings, moisture variations, and acidic air conditions. The degradation mechanisms of glazed components under combined environmental stresses and the efficacy of hybrid reinforcement systems are still poorly understood, despite earlier research examining deterioration processes in ceramic and glazed materials and exploring various conservation strategies. Therefore, to promote the scientific protection of historic glazed architectural features, rigorous research into deterioration mechanisms and suitable conservation measures is required.

The low melting point of lead-glazed items is one of their distinguishing characteristics. Glazed components from the Qing Dynasty usually contained more than 50% lead oxide, and the glaze melted between 750°C and 950°C. Coal gangue, which ranges in SiO2 concentration from 37% to 68% and Al2O3 content from 11% to 36%, is the main raw material used to make glazed tiles. The body is usually burned at temperatures higher than 1000°C to achieve adequate sintering [2–4]. A two-stage kiln firing technique is required due to the high firing temperature of the body and the low melting point of the glaze. To achieve sintering, the body is first burned at temperatures higher than 1000°C. The glaze is fired below 950°C in the second step, where it melts and forms a link with the body. Throughout ancient China, especially in the north, glazed tiles were extensively utilized in the construction of palaces, temples, and other important buildings [5,6]. The colors of glazed tiles are subject to stringent standards; typical colors include yellow, green, blue, black, and purple. The highest-grade color, yellow, is only used for imperial palaces and tombs, such Beijing’s Ming Tombs and the Forbidden City. Purple and black tiles were mostly used in royal gardens for pavilions and buildings, whereas green and blue tiles were utilized in royal homes or by high-ranking officials (only by imperial decree).

Research suggests that flaws in the fire process of glazed tiles are a major internal component in the causes of glaze peeling and cracking. The glaze surface will unavoidably develop a large number of cracks during burning. These fissures become significant corrosion channels for the glazed tiles during environmental deterioration because they lower the density of the glaze layer and the bonding between the body and the glaze. A ceramic body and a low-temperature lead glaze make up glazed tiles. The glaze layer is a lead-silicon glass phase, whereas the body is composed of crystalline phases and a tiny quantity of molten glass phase. The two have very different coefficients of thermal expansion due to the variances in their form and composition [7]. During burning and cooling, this discrepancy in thermal expansion coefficients causes stress in the glazing layer, which decreases its adherence and results in peeling and cracking. Furthermore, because of the inconsistent thermal expansion coefficients, abrupt variations in the surrounding temperature can also put stress on the body and glaze, which can eventually cause the glaze to peel or shatter. Changes in the external environment play a significant role in the deterioration of glazed tiles, in addition to internal causes. Glaze peeling was identified as the main issue after the Palace Museum’s glazed tiles were examined for damage. The primary causes of glazing peeling were variations in water and environmental temperature, particularly below-freezing temperatures. Significant variations in the outside temperature and humidity led to glaze peeling, according to simulated cyclic experiments conducted on the Hall of Mental Cultivation’s glazed tiles. Additionally, when glaze thickness increased and the body’s and glaze’s thermal expansion coefficients differed, the rate of glaze peeling increased. It was also hypothesized that the main causes of glaze peeling on historic building glazed tiles were high summer temperatures and heavy precipitation. In addition to harming the glaze, changes in the external environment can also result in fractures and cracks in the tile body [8].

In the past, composite materials like Remmers 300 and materials like Primal SF, Paraloid B72, and acrylates were frequently utilized. Furthermore, hydrophobic nanoparticles such as hydroxyl-terminated polydimethylsiloxane (PDMS) and tetraethoxysilane (TEOS) [9], used in stone restoration in conjunction with nonionic surfactants Protective coatings for building surfaces have been made using a variety of modified ethyl silicate consolidants, organosilane consolidants, copolymers of fluorinated acrylates, methacrylates, and vinyl ethers, as well as polyhydroxybutyrate and poly-L-lactide. Furthermore, many conservators also select new materials. For instance, hydrophobic organic silicon resins (SIC-3) and silicon-based compounds containing fluorine (SIC-1 and SIC-2) have been selected to solve glaze shedding and damage to glazed tiles in old buildings. On the surface of glazed tiles, these materials may deteriorate over time and exacerbate damage if they are not cleaned right away.

Recent studies on glazed-tile and ceramic-heritage conservation can be summarized into three research streams. First, archaeometric studies have clarified the raw materials, firing technology, glaze-body interface, and decay processes of Chinese architectural glazed tiles, providing a material basis for conservation decisions [10]. Second, protective-treatment studies have demonstrated that silicon-based hydrophobic agents can reduce glaze shedding and water-induced deterioration, but their compatibility, penetration depth, surface color change, and long-term stability must be carefully evaluated before use on immovable heritage components [9]. Third, sol-gel organic-inorganic hybrid systems based on TEOS and PDMS-OH have been shown to form Si-O-Si networks, improve hydrophobicity, and enhance the mechanical consolidation of porous silicate or ceramic substrates while maintaining relatively small color changes [11,12]. However, most existing work focuses either on material characterization or on single protective treatments. A systematic connection among field deterioration patterns, accelerated environmental simulation, quantitative performance evaluation, and microscopic reinforcement mechanisms for Shanxi glazed architectural components remains insufficient. This gap defines the main contribution of the present study.

Research on the precise types of damage to glazed tiles in outdoor settings is still lacking, despite experimental investigations having been carried out [13–15]. A comprehensive field survey in Shanxi will be part of this project, methodically identifying various forms of glazed tile degradation. The study will concentrate on actual damage to glazed components, analyzing local use patterns using visual observation and anthropological techniques, and provide a thorough explanation of the different kinds of damage to these tiles. The deteriorating characteristics of glazed tile components used in traditional Chinese architecture under environmental pressures such temperature fluctuation, humidity change, and acidic environments are comprehensively investigated in this work. Additionally, to increase the structural integrity of degraded glazed tile bodies, reinforcement techniques based on hydroxyl-terminated polydimethylsiloxane (PDMS–OH) and tetraethyl orthosilicate (TEOS) are assessed. This work attempts to shed light on the efficiency of reinforcement as well as the underlying mechanisms by integrating macroscopic performance evaluation with microscopic characterization techniques such as scanning electron microscopy (SEM) and X-ray photoelectron spectroscopy (XPS). The findings offer a scientific foundation for the preservation and repair of glazed architectural elements in old structures. The research is divided into three primary phases in order to accomplish these goals. First, macroscopic observation and material characterisation are used to examine the deteriorating characteristics of glazed tile components exposed to environmental stress factors. Second, TEOS/PDMS–OH systems with various formulations are used in reinforcement studies to assess their efficacy in enhancing the mechanical and durability characteristics of the glazed tile body. Third, the reinforcing mechanism and the interaction between the reinforcement materials and the ceramic matrix are examined through microscopic investigations utilizing SEM and XPS.

## 2. Research locations

One of the important Chinese provinces that is well-known for its usage of colorful glaze in architecture is Shanxi Province. The abundance of coal resources in Shanxi and the existence of “ganzi soil,” which is necessary for firing colorful glaze, are the main causes of this. The famous architectural sites of the ancient city of Datong and the colorful glaze complex in Jiexiu are two notable examples of colored glaze architecture in Shanxi. Due to their excellent construction standards, the roofs of these old buildings were typically composed of colored glazed tiles. Additionally, a lot of structures had memorial arches and screen walls made of colorful glaze [16]. To record the state of preservation of glazed architectural elements, a preliminary field assessment was carried out at several historic sites in Shanxi Province. Ten exemplary historic buildings were chosen for in-depth examination from this larger assessment based on factors such historical value, the structural integrity of glazed components, and documentation accessibility. To gather typical observations of deterioration patterns, several glazed components (such as wall elements, roof ridges, and decorative decorations) situated in various structural positions were analyzed for each building. This study primarily examines the deterioration of colored glaze components in ancient buildings across Taiyuan, Jincheng, Datong, Jiexiu, Yangcheng, and other locations in Shanxi Province, encompassing the entire region. All surveyed buildings are classified as National Key Protection Units. Detailed information about the buildings is shown in Table 1.

**Table 1:** Overview of glazed tile components in architectural research.

number	Building name	Coloured glaze component position	Location	Construction Time	Total Components Inspected	Roof Tiles (%)	Wall Tiles (%)	Decorative Ornaments (%)	Surface Cracking (%)	Glaze Pulverization (%)	Color Fading (%)	Local Corrosion (%)
1	Chongshan Temple	Glazed tile	Taiyuan City	1391	35	40	35	25	15	20	30	5
2	Houtu Temple	Glazed tile	Jiexiu city	457	28	42	33	25	18	15	25	7
3	Taihe rock colored glass memorial archway	Coloured glaze archway	Jiexiu city	1897	32	38	37	25	12	22	28	8
4	XianshenTemple	Glass wall	Jiexiu city	1055	30	40	35	25	14	18	27	6
5	Shousheng Temple and the colored glass tower	Coloured glaze tile	Yangcheng County	1608	25	36	36	28	16	20	26	5
6	HaiHuitwo towers	Coloured glaze tile	Yangcheng County	894	33	41	34	25	15	19	29	7
7	Guqinglian Temple	Glazed tiles	Jincheng city	559	27	39	36	25	14	21	28	6
8	Shanhua Temple	Glass wall	Datong City	1445	30	40	35	25	13	18	27	6
9	Datong House Confucian Temple	Glass wall	Datong City	1375	28	42	33	25	15	19	26	5
10	Guanyin TANG	Glass wall	Datong City	Qing dynasty	35	40	35	25	16	20	30	6

## 3. Methods

In-situ detection is required because the surveyed colored glaze components are immovable cultural artifacts. This study prioritizes non-destructive testing techniques in accordance with cultural heritage protection standards. The GB/T 3810 standard for residential ceramic tiles was followed in the on-site evaluation of the colored glaze’s quality. Surface flaws, degradation, hardness, gloss, and color fluctuation of the colored glaze were all examined scientifically [17]. Every performance test was carried out on a minimum of three replicate specimens under identical conditions to guarantee the validity of the experimental findings. To assess the stability of the measurements, the average value and standard deviation were computed. A number of quantitative metrics, such as improved mechanical strength, surface integrity, and resistance to environmental deterioration, were used to assess the reinforcing treatment’s efficacy. Compressive strength measurements were used to evaluate mechanical performance, while SEM views were used to analyze microstructural integrity. Additionally, the long-term protective efficacy of the reinforcement materials was assessed using durability indicators including water resistance.

### 3.1. Quantitative evaluation criteria for degradation and reinforcement effects

To improve the transparency of the experimental methodology, the degree of degradation and the effectiveness of reinforcement were evaluated using a unified indicator system. The degradation level was assessed from four aspects: (i) mechanical deterioration, represented by Shore hardness and Leeb hardness loss; (ii) surface integrity, represented by the proportion of visible cracks, glaze peeling, pores, and powdering observed by camera, handheld microscopy, and SEM; (iii) color and gloss variation, represented by the CIE Lab color difference (ΔE) and gloss retention; and (iv) durability, represented by water contact angle (WCA), water absorption rate (WAR), penetration depth, and surface water-resistance performance. For an indicator that decreases during deterioration, the degradation ratio was calculated as follows:

where X₀ is the initial value and X_t_ is the value after the aging or corrosion treatment. For an indicator that increases during deterioration, such as WAR or ΔE, the degradation ratio was calculated as follows:

The reinforcement efficiency for a positive indicator was calculated as follows:

where Xᵈ and Xʳ denote the degraded and reinforced values, respectively. For negative indicators, such as water absorption or color difference, the reinforcement efficiency was calculated as follows:

A larger D value indicates more serious deterioration, whereas a larger R value indicates a better reinforcement or repair effect.

The interpretation criteria were defined as follows. For color compatibility, ΔE < 3 was considered visually acceptable, 3 ≤ ΔE < 5 indicated slight but perceptible change, and ΔE ≥ 5 indicated obvious chromatic change. For mechanical reinforcement, an increase in Leeb hardness greater than 10% was considered effective consolidation, while a small change in Shore hardness was interpreted as evidence that the consolidant did not form a thick surface film. For durability, lower WAR, higher WCA, and deeper but stable penetration depth indicated better resistance to moisture migration. Surface integrity was evaluated by comparing microscopic images before and after treatment; reduced pore connectivity, fewer loose particles, and improved particle bonding were considered evidence of effective matrix consolidation. All quantitative measurements were performed on at least three replicate specimens, and the results were expressed as mean ± standard deviation when applicable.

(1) A Dino-Lite AM3111 handheld microscope was used to observe the vibrant glaze. This instrument allows for intimate observation of glazing fractures and other deterioration events and is ideal for on-site inspection due to its tens to hundreds of times magnification [18,19].
(2) The microstructure and chemical composition of the materials were investigated using an ambient scanning electron microscope (Thermo Fisher Scientific Quattro S) equipped with a Bruker QUANTAX EDS X-ray energy dispersive spectrometer. The accelerating voltage was set between 15 and 20 kV, the working distance was around 10 mm, and the low vacuum was 50 Pa. The pictures were taken using the CBS backscattered electron method. The cross-sectional sample was polished and ground after being submerged in epoxy resin.
(3) The physical phase composition of the materials was investigated using a Renesas Vavia laser micro-focusing Raman spectrometer. This apparatus is equipped with a research-grade Leica microscope that has a spatial resolution of less than 0.5 μm. The experimental settings included an excitation wavelength of 532 nm, a laser power of 280 mW, a laser power density of 1.0%, a scan duration of 10 seconds, and a total of ten scans.
(4) The colored glaze and the exposed body were tested for hardness using a Leeb hardness tester. Despite the relative durability of the tinted glaze, care must be taken during the test to avoid further damage. The test must be continuously observed, and it must immediately stop if harm is discovered. The gloss of the colored glaze was measured using a gloss meter. Because some colored glazes may have curved surfaces, a smaller measurement aperture is recommended. Furthermore, because varying gloss levels might cause some areas to have considerable shine and others to have no sheen at all, a test angle of 60° is usually sufficient.
(5) The color of the tinted glaze was evaluated using a colorimeter. The Lab value, which was created by the International Commission on Illumination (CIE) based on how people perceive color, was used for the measurement. Averaging the data of multiple test sites yielded the representative value [20]. Color difference testing was applied to components of different colors and positions within the same structure. Significant variations show discoloration brought on by lesions, but a certain amount of color variability is acceptable.
(6) Surface water resistance test. To evaluate the water resistance performance of the reinforced glazed tile body, a surface water absorption test was conducted. The treated and untreated specimens were immersed in distilled water for a specified period under controlled temperature conditions. The mass variation before and after immersion was recorded to determine the water absorption rate. The water resistance of different reinforcement formulations was compared based on the reduction in water absorption and the stability of the specimen surface.

## 4. Damage to the glazed tile enamel on-site

Colored glaze deterioration can be divided into two categories: production-related deterioration and usage-related uncontrolled variables. Both types of deterioration can have a substantial impact on the integrity of colored glaze components [21].

### 4.1. Glass glazing that is inferior because of the manufacturing process

**Fig 1:**
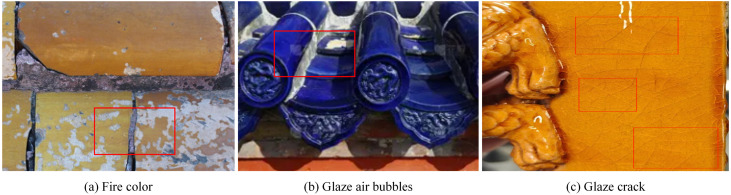
Deterioration of glazed tiles caused by manufacturing processes: (a) fire color; (b) glaze air bubbles; (c) glaze crack.

“Fire color,” which appears on the surface of glazed components during the firing stage, is a common production flaw. This phenomenon happens when the kiln’s high internal pressure pushes flames in the direction of lower pressure zones [22], creating a flame flow that hits the glaze surface and creates the fire color depicted in Fig 1a. Bubbles appear on the glaze surface as a result of the oxides in the clay body and the thick glaze layer releasing gasses during high-temperature burning. “Bubble pits” occur when the glaze cannot completely melt and level due to insufficient heat preservation time (Fig 1b). Glaze cracking is another common flaw (Fig 1c). The glaze stone progressively liquefies when the glaze turns molten during firing. Surface fractures result from the glaze’s thermal expansion coefficient exceeding the ceramic body’s when the kiln cools, reducing its adherence. During the components’ later service life, these fissures may hasten deterioration [23].

### 4.2. The glass glaze used naturally is of inferior quality

**Fig 2:**
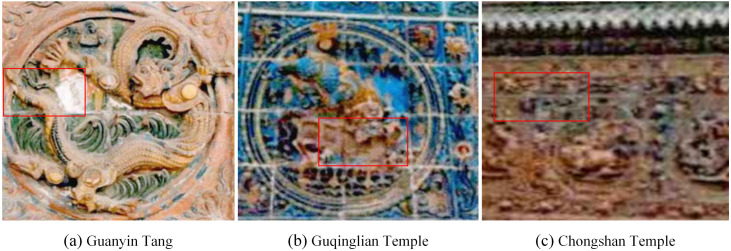
Vegetation-related deterioration observed at representative sites: (a) Guanyin Tang; (b) Guqinglian Temple; (c) Chongshan Temple.

The most popular colored glaze components are glazed tiles, which are usually placed on building roofs to prevent rainwater intrusion [24,25]. However, they are also vulnerable to harm from vegetation growth due to their roof placement. Representative vegetation-related deterioration observed at typical sites is summarized in Fig 2.

**Fig 3:**
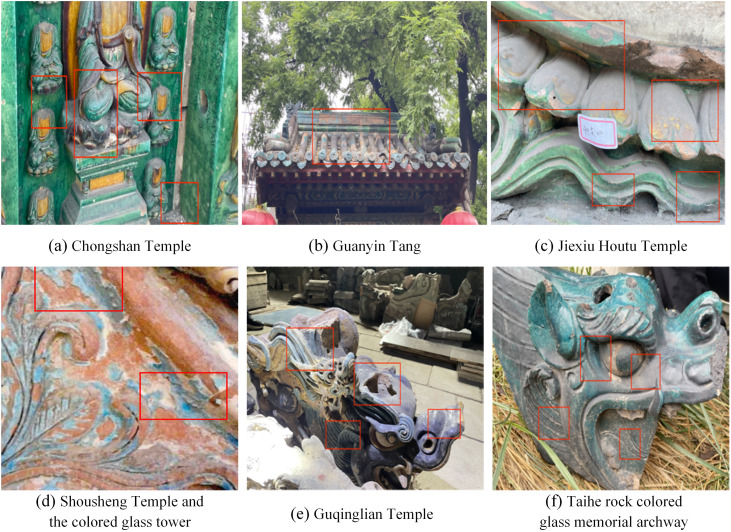
Dust accumulation deterioration observed at representative sites: (a) Chongshan Temple; (b) Guanyin Tang; (c) Jiexiu Houtu Temple; (d) Shousheng Temple and the colored glass tower; (e) Guqinglian Temple; (f) Taihe rock colored glass memorial archway.

Dust progressively builds up in surface indentations and sticks firmly to the glaze layer when colored glaze components are left outside for extended periods of time without routine cleaning. The deposited dust may be washed away by severe weather events like storms, torrential rain, or snowfall. This process may also cause the glazing surface to partially peel. Representative examples of dust accumulation deterioration are presented in the corresponding figure (Fig 3).

**Fig 4:**
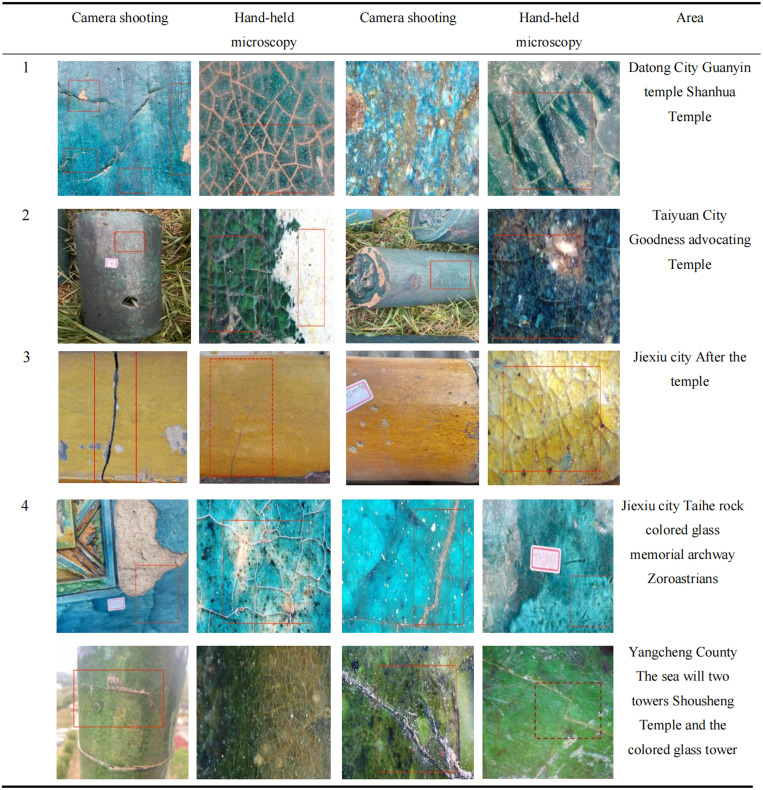
Glaze cracking deterioration observed by camera and handheld microscopy at representative sites in Datong, Taiyuan, Jiexiu, and Yangcheng.

Throughout the service life of colored glaze components, cracking and partial glaze layer loss are common [26]. These flaws are frequently linked to crazing that develops throughout the production process. The components repeatedly expand and contract over prolonged use due to daily and seasonal temperature fluctuations, which progressively exacerbates the initial microcracks. The growth and present state of these fissures are visible under a handheld microscope, suggesting that the spread of production-induced crazing is a significant contributing element to this degradation. Furthermore, the presence of salt crystals close to the fissures suggests that capillary movement within the cultural relic materials is responsible for the migration of soluble salts, which is linked to glazing degradation. Representative field and microscopic observations of glaze cracking deterioration are shown in the corresponding figure (Fig 4).

## 5. Analysis of the causes of degradation

### 5.1. The effects of temperature and humidity changes

**Fig 5:**
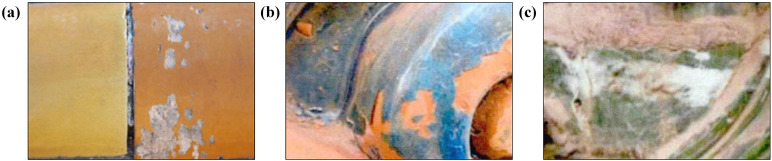
Glaze peeling observed on the HTWS samples: (a) edge peeling of the yellow glaze; (b) surface peeling of the blue glaze; (c) surface peeling of the green glaze.

The environmental requirements for the deterioration of glazed tiles were identified based on the examination of the climate and preservation environment of glazed tiles in Chapter 4 [27]. Temperature, wetness, and abrupt changes in the surroundings were among them; the experimental circumstances included abrupt shifts between high and low temperatures. These settings included 24 hours of room temperature immersion followed by 8 hours of low temperature immersion at −30°C (WSLT) and 8 hours at 150°C followed by 24 hours of warm water immersion (HTWS). These elements mostly consist of temperature, moisture, and abrupt changes in the surrounding environment. As a result, the design of the experiment mimicked sudden changes in temperature. Immersion at room temperature for 24 hours, followed by 8 hours at −30°C (WSLT), and exposure to 150°C for 8 hours, followed by 24 hours in warm water (HTWS) were the test conditions.The environmental simulation studies’ temperature settings were designed to hasten the degradation processes that usually take place after extended contact to the environment. Accelerated aging studies are frequently used to replicate long-term environmental impacts within a constrained experimental timescale, even though these temperature levels may beyond typical short-term climatic circumstances.For every experimental group, three parallel glazed tile specimens with dimensions of 2.5 cm × 3 cm × 2 cm were created. Throughout the trial, the samples’ performance and visual state were routinely noted, with special focus on damage to the ceramic body and glaze layer [28,29]. In addition to expanded glaze fissures and localized glaze peeling, the WSLT samples showed body fractures and cracking. The HTWS samples, on the other hand, had no obvious bodily damage, albeit there was some slight glaze flaking around the borders. Figs 5 and 7 show the HTWS and WSLT samples’ comprehensive morphologies.

**Fig 6:**
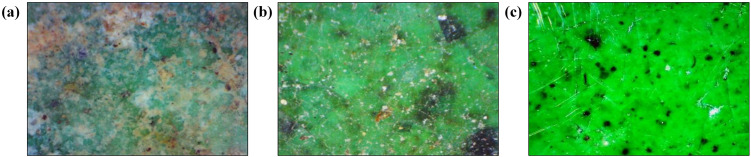
Crack patterns of WSLT samples: (a) cracks in the green glaze; (b) additional cracks in the green glaze; (c) central peeling of the green glaze.

The selection of 150 °C and −30 °C was intended as a stress-screening accelerated aging protocol rather than a direct reproduction of ordinary outdoor temperature fluctuations. The high-temperature stage was used to intensify the thermal-expansion mismatch between the lead-silicate glaze and the ceramic body, while the low-temperature stage was used to amplify moisture-related freeze-thaw stress in pores, cracks, and the glaze-body interface. The alternating immersion and thermal stages were therefore designed to highlight potential failure pathways, including crack propagation, glaze debonding, pore expansion, and moisture-driven matrix weakening. This design follows the general principle of accelerated aging in conservation science, where elevated environmental stresses are applied to reveal possible degradation tendencies within a limited experimental period [30]. Nevertheless, the parameters should be interpreted as comparative laboratory conditions, and the resulting damage does not necessarily correspond one-to-one to the slow natural weathering experienced by historical buildings over several hundred years.

Due to its lower thermal expansion compatibility, the yellow glaze, which comprises iron oxides and lead-based fluxes, shows more noticeable microcracking during thermal cycling. In contrast, when exposed to acidic conditions, the green glaze enhanced with copper ions exhibits higher localized corrosion but slower surface powdering. The different deterioration patterns seen between the two glazing types are explained by the unique variances in microstructural damage and corrosion product production revealed by SEM and XPS data.

The little patches of glaze peeling that developed on the green and yellow glazes of the HTWS samples following ninety test cycles are depicted in Fig 5. The porous nature of the underlying body showed considerable water absorption after the glaze came off. Because the bond between the glaze and body weakens at the borders, peeling is likely to spread along the original problem areas. Different levels of body deterioration and glazing were seen in the WSLT samples [31]. Cracks decreased the glaze’s surface density in addition to interfering with the glaze’s ability to stick to the body. As seen in Fig 6, the tiny “ice cracks” progressively joined together during degradation to develop huge fissures that almost completely covered the glaze surface.

As shown in Table 2, the water contact angle increased and the water absorption rate decreased after TEOS/PDMS−OH treatment, indicating improved surface water resistance.

**Table 2:** Surface water resistance.

Sample	TEOS:PDMS−OH Ratio	WCA (°)	WAR (%)
Control (untreated)	–	42.5 ± 1.2	12.8 ± 0.6
Sample A	3:1	78.3 ± 1.5	6.2 ± 0.3
Sample B	1:1	82.1 ± 1.4	5.8 ± 0.4

**Fig 7:**
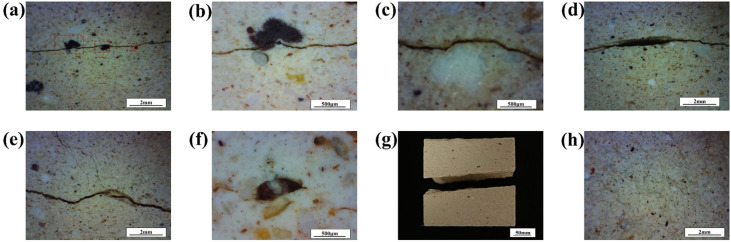
Morphology of HTWS specimen carcass failure: (a), (b), (c) Morphology of cracks and large particles at cracks; (d) Morphology of cracks and pores; (e) Morphology of interwoven cracks; (f) Morphology of pores; (g) Fracture morphology; (h) Fracture cross section.

The data clearly demonstrate that TEOS and PDMS−OH reinforcement significantly improves the water resistance of the glazed tile components. This addition provides a quantitative basis for assessing the durability of the reinforced samples and complements the mechanical property evaluation. Microcracks initially emerged in the WSLT sample matrix during degradation. The length and diameter of these fissures progressively grew as deterioration persisted. As seen in Fig 7, a significant crack eventually spread across the matrix, causing it to fail. Because of the uneven thermal expansion between the particles and the surrounding matrix, cracks tended to form along the edges of big particles [32]. Additionally, pores served as preferred locations for corrosion, and as deterioration advanced, pore areas grew, creating tiny fissures along their edges. The cross-sectional particles became more porous and prone to detachment during further degradation once the matrix broke. According to experimental findings, abrupt temperature changes or extremely high or low temperatures had little effect on the environmental deterioration of glazed tiles, whereas moisture was a major factor. While the WSLT cycles contributed to matrix cracking and the enlargement of glaze cracks, the HTWS treatment primarily generated localized peeling close to the glaze margins.

### 5.2. Changes in glaze morphology before and after acid rain corrosion

**Fig 8:**
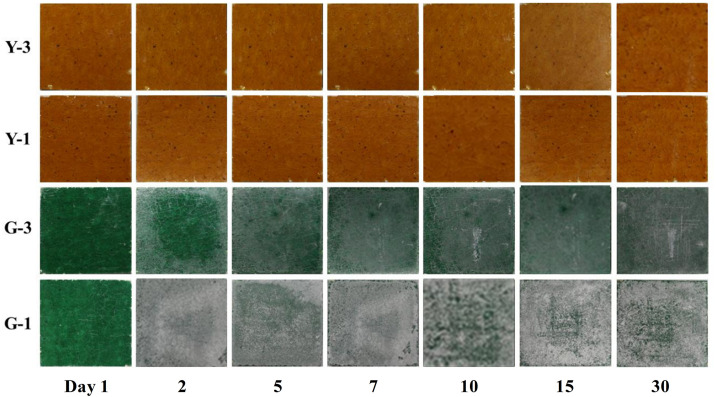
Changes in the macroscopic morphology of the glazed tile surface under acid rain corrosion over 30 days.

Macroscopic Morphology: Fig 8 displays the glazed tiles’ macroscopic surface morphology following 30 days of exposure to acid rain. Yellow and green glazes showed different corrosion tendencies. Under acid rain simulations at pH 3 and 1, the yellow glaze showed no discernible corrosion damage. On the other hand, depending on the pH of the corrosion solution, the green glaze displayed notable variations in surface morphology [33]. White corrosion products stuck to the green glaze’s edges after a day in the pH 3 solution. The corrosion layer on the glaze surface stabilized and thickened throughout the course of five days. It was challenging to remove the corrosion layer by the 30th day since it was comparatively dense and well-adhered. As shown in Fig 8, the quantity of surface corrosion products rose on the third and fifth days following partial removal, but did not significantly alter over the remaining corrosion period. The green and yellow glazes displayed different corrosion behavior under the same circumstances: the green glaze was almost entirely corroded, whereas the yellow glaze showed no discernible deterioration.

**Fig 9:**
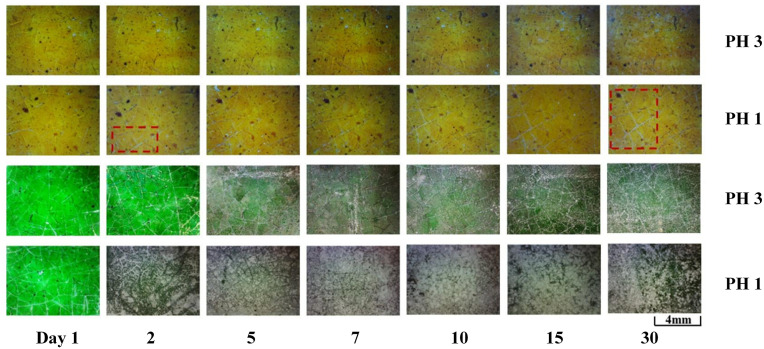
Changes in the glaze surface morphology of glazed tiles under acid rain corrosion over 30 days (via video microscope).

Video Microscopic Morphology: A microscope was used to study the microscopic morphology of the glazed tile surfaces during corrosion; the findings are displayed in Fig 9. The yellow glaze showed no discernible surface alterations after being exposed to a pH 3 solution for 30 days. After a day, a tiny quantity of white corrosion product developed inside the glaze fractures in the pH 1 corrosion solution [34]. After 30 days, almost every crevice in the studied area was filled with corrosion particles due to the progressive increase in corrosion products caused by the prolonged exposure. After a day in the pH 3 solution, the green glaze showed very little surface deterioration. Both inside fissures and over a large portion of the surface, corrosion products gathered. After just one day, a significant amount of loose, white, granular corrosion products covered the glazing in the pH 1 solution, and the corrosion morphology stayed mostly unaltered for the next thirty days.

**Fig 10:**
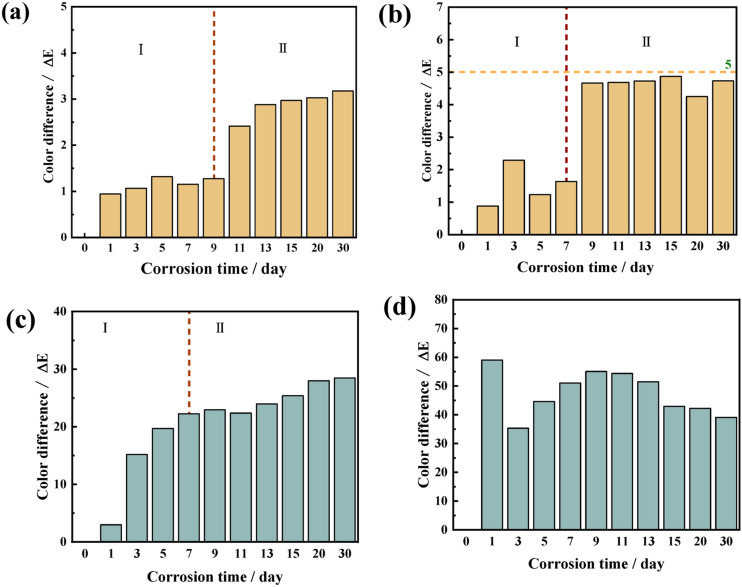
Color differences (ΔE) of glazed tiles under acid rain corrosion over 30 days. (a) Yellow glaze, pH = 3; (b) Yellow glaze, pH = 1; (c) Green glaze, pH = 3; (d) Green glaze, pH = 1. ΔE values represent the overall color change compared with the initial uncorroded samples, allowing quantitative assessment of deterioration under different pH conditions.

Color: The observed corrosion behavior was reflected in the color difference values of the green and yellow glazes before and after corrosion, which varied significantly (Fig 10). Color difference values for the yellow glaze stayed below 5, suggesting that the modest amount of corrosion products had little effect on the glaze’s appearance [35]. Over time, the yellow glaze’s color difference grew in the pH 3 acid rain solution. Changes were negligible from day one to day nine, but from day nine to day thirty, the values significantly increased, indicating moderate discoloration. After a day, a significant buildup of white corrosion products developed on the surface of the pH 1 solution, leading to a color difference value as high as 60, which is regarded as serious discoloration. Surface corrosion products were eliminated before to immersion on the third day in order to better examine the effect; following this, the overall color difference remained high and grew over time. The loose nature of the surface corrosion products, which could readily separate, contributed to variations in the color difference measurements.

**Fig 11:**
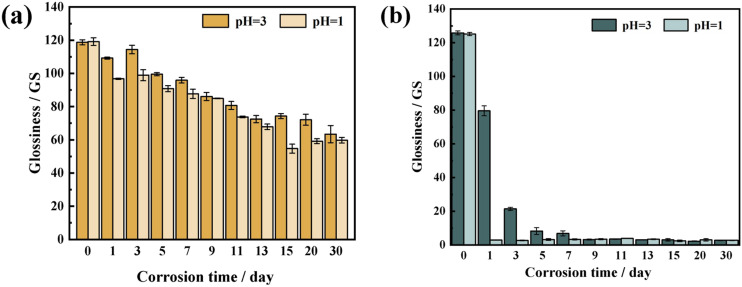
Changes in surface gloss of glazed tiles under acid rain corrosion over 30 days. (a) Yellow glaze; (b) Green glaze. Gloss retention (%) indicates the percentage of initial gloss remaining after treatment, providing a quantitative measure of surface deterioration for each sample.

Gloss: The gloss alterations of glazed tile surfaces following a 30-day acid rain simulation are depicted in Fig 11. The shine of the green and yellow glazes progressively diminished as corrosion advanced. In the pH 1 solution, the yellow glaze’s shine was reduced more than in the pH 3 solution. A discernible decrease in yellow glaze gloss started around day nine, as seen in Fig 11(a). The gloss loss was 46.58% for pH 3 and 49.82% for pH 1 by day 30. During the corrosion stage, the green glaze saw a more noticeable loss of sheen. After just one day, the green glaze in the pH 3 solution lost 36.72% of its sheen. These results indicate that, under the same acidic conditions, the green glaze is more susceptible to gloss loss than the yellow glaze.

## 6. Reinforcement of glazed tile matrix by PDMS−OH/TEOS

According to field assessments, the majority of the glazed tiles found in existing old buildings have varied degrees of deterioration [36]. This study divides the locations of glazed tile damage into two categories: glaze layer and body, using temperature and humidity variations as well as acid rain corrosion simulation studies. Among these, glaze repair and glaze re-firing are the primary methods for protecting the glaze layer [10]. The protection of glazed tile bodies, specifically the materials used to safeguard existing glazed tile bodies, is the primary focus of this article. The service life of glazed tiles can be further increased by enhancing the body’s mechanical and waterproof qualities. Tetraethyl orthosilicate (TEOS) is the main reinforcing material. It is modified with hydroxyl-terminated polydimethylsiloxane (PDMS−OH). Ethanol is used as solvent to investigate the effects of PDMS−OH and ethanol content on the reinforcement effect. By comparing the water absorption rate, mechanical properties, and water resistance of the body after reinforcement, the formula with the best reinforcement effect is selected [37].

Before reinforcing, the glazed tiles were subjected to a simulated degradation treatment based on the simulation results in Chapter 5. For three hours, the sample substrate was dried in an oven set at 105°C. It was immediately removed and submerged for three hours in a pH-3 acid rain simulation solution. Lastly, it was frozen for three hours at −18°C. Specific masses of TEOS, PDMS-OH, and ethanol were weighed and carefully combined using an analytical balance. The glazed tile substrate sample was covered with equal amounts of reinforcing material. The sample was put in a fume hood to allow the reinforcing material to fully penetrate after making sure all surfaces were sufficiently moistened. Finally, it was placed in a 35℃ vacuum drying oven for 12 hours and then left at room temperature for 3–4 days before various performance tests were conducted.

To directly address the main degradation mechanisms identified in our study, the PDMS–OH/TEOS reinforcement was designed with specific functional properties: the hydrophobicity of PDMS–OH reduces water absorption in the tile body, limiting expansion and cracking caused by freeze-thaw cycles; the TEOS component forms a silica network, enhancing the structural integrity of the glaze and body; together, the PDMS–OH/TEOS matrix acts as a protective barrier, mitigating acid penetration and slowing chemical corrosion under acidic conditions. In addition, the combined system improves cohesion between the glaze and tile body, strengthening the mechanical bond and reducing powdering under environmental stress. This mechanistic rationale explicitly links material selection to the key failure modes observed in the field and laboratory, providing a clear foundation for the proposed conservation strategy.

### 6.1. Color difference compatibility analysis

**Fig 12:**
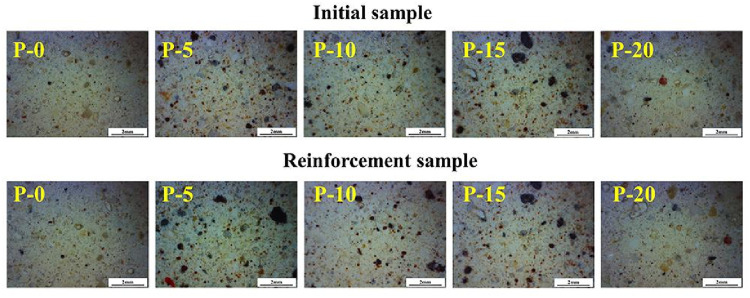
Comparison of morphology before and after reinforcement.

**Fig 13:**
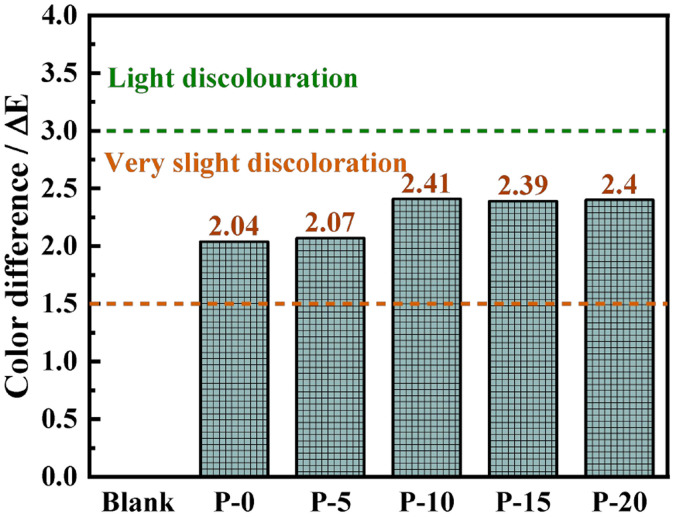
Color difference values of tile body reinforced with different PDMS−OH contents.

Maintaining a consistent style is crucial for the restoration and preservation of cultural artifacts, and treated samples should have colors that are comparable to those of their original state. The morphology of the samples both before and after the reinforcing material was applied is depicted in Fig 12. The overall appearance was not significantly affected by the treatment. Without creating a discernible layer on the surface, the reinforcing agent effectively penetrated. The surface color changes following reinforcing are shown in Fig 13. When compared to samples reinforced with pure TEOS, samples treated with PDMS−OH showed somewhat larger color difference values, which increased gradually as the PDMS−OH level increased. However, compared to the original glazed tile surface, the overall color change was negligible, with color difference values ranging from 1.5 to 3, suggesting very mild deterioration that stayed within the permitted range [38].

### 6.2. Hardness

**Fig 14:**
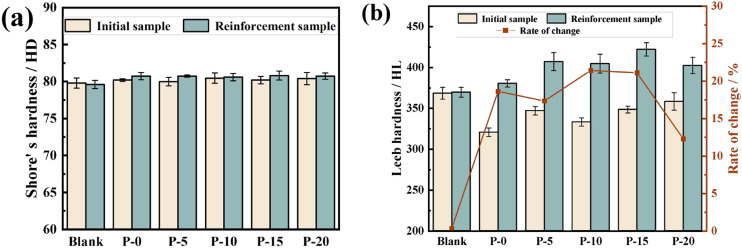
Shore hardness, Leeb hardness, and matrix change rate of samples with varying PDMS-OH concentrations. (a) Shore hardness; (b) Leeb hardness.

The shore hardness of specimens supplemented with TEOS and PDMS-OH/TEOS is shown in Fig 14(a). The values of the unreinforced specimens varied from 79.8 HD to 80.5 HD. The average hardness of the unreinforced specimens was 80.2 HD, whereas the TEOS-reinforced specimens had a slightly higher hardness of 80.7 HD. After adding PDMS-OH, a comparable improvement in hardness was noted. The main purpose of shore hardness testing is to describe how hard the tile body surface is. The reinforced specimens’ surface hardness value did not significantly alter after reinforcement, indicating that the reinforcing material had high general permeability and did not accumulate excessively on the surface [39].

The Leeb hardness values of the TEOS and PDMS−OH/TEOS-reinforced specimens are displayed in Fig 14(b). The Leeb hardness values of the tile body before and after reinforcement were tested for each specimen. In comparison to the unreinforced specimens, the rate of change was computed [40]. The Leeb hardness of the TEOS-reinforced specimens increased by 18.63%. The Leeb hardness first rose and subsequently slightly decreased for specimens containing PDMS−OH. Of them, sample P-10 showed the largest rise (21.4%), closely followed by another sample (21.1%), and sample P-20 showed the lowest increase (12.3%). Leeb hardness testing mainly shows the samples’ total reinforcing effect. The reinforcement material PDMS−OH/TEOS significantly improved the mechanical properties of the tile body. PDMS−OH addition levels of 10% and 15% yielded better results.

### 6.3. Surface permeability analysis

**Fig 15:**
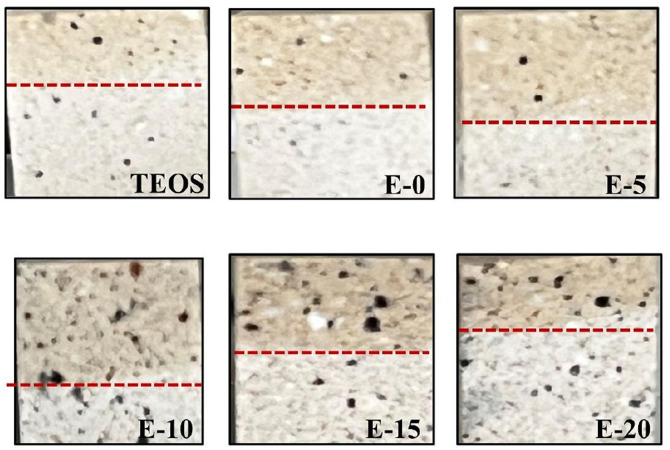
Penetration morphology of various reinforcement materials.

The glazed tile matrix’s porous structure facilitates the efficient penetration of reinforcing elements. The depth of each reinforcing material was evaluated in order to evaluate the impact of ethanol on penetration; the findings are shown in Fig 15 and Table 3. The penetration depth of the TEOS-reinforced sample was approximately 3 mm. The depth rose to 6 mm with the addition of PDMS−OH, which is about twice as deep as the TEOS-only sample. When the ethanol content was changed in the PDMS-OH/TEOS system, penetration depth first rose and then fell. At 10% ethanol, a maximum depth of 9.5 mm was recorded, which is around 3.2 times deeper than the TEOS sample and 1.6 times deeper than the E-0 sample. The penetration depth dramatically dropped above 15% ethanol. According to these findings, adding ethanol and PDMS-OH improves TEOS penetration [41], however the ethanol content shouldn’t be more than 15%. 10% ethanol produced the best penetration and overall reinforcing impact in the PDMS−OH/TEOS system, including water absorption, surface water resistance, and mechanical properties.

**Table 3:** Penetration depth of each reinforcement materials.

Sample Number	Sample Composition	Ethanol Addition (wt%)	Penetration Depth (mm)
TEOS	TEOS	0	3
E-0	PDMS−OH /TEOS	0	6
E-5	PDMS−OH /TEOS	5	7.5
E-10	PDMS−OH /TEOS	10	9.5
E-15	PDMS−OH /TEOS	15	7
E-20	PDMS−OH /TEOS	20	4.5

The findings show that as compared to the untreated specimens, the reinforced samples showed noticeably better water resistance. The optimized TEOS/PDMS–OH ratio demonstrated the lowest water absorption rate among the evaluated formulations, indicating that the reinforcing treatment successfully decreased the porosity of the glazed tile body and improved its resistance to moisture penetration.

### 6.4. Micromorphological analysis

**Fig 16:**
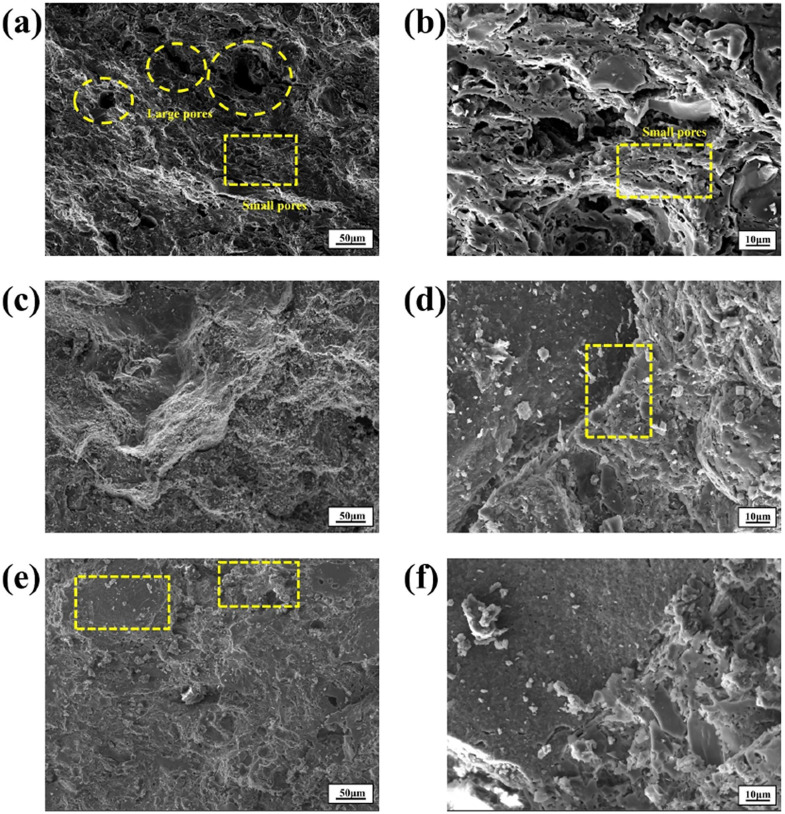
Microstructure of Blank, P-0, and E-10 samples: (a) and (b) Blank sample; (c) and (d) P-0 sample; (e) and (f) E-10 sample.

The samples’ shape and microstructure are shown in Fig 16. With many internal and external pores, the glazed tile matrix has a porous structure. Both big and tiny pores were present in the interparticle network, and pore size increased and particle connection reduced after environmental deterioration [42]. Following reinforcement, samples P-0 and E-10 showed a significant decrease in pore diameters as the reinforcing material partially filled the pores, creating a smoother matrix surface. This implies that certain particles were attached to by the reinforcing substance, a characteristic that is more noticeable at higher magnifications. Accordingly, the reinforced samples’ water absorption dramatically dropped, which is in line with surface water resistance tests and microstructural observations. There are two primary reasons for this improvement: first, the reinforcing material filled pores and bonded with particles; second, the matrix surface became hydrophobic, preventing water infiltration. In general, reinforcing improved mechanical characteristics, densified the structure, and decreased matrix porosity.

In conclusion, PDMS−OH/TEOS reinforcing materials successfully improve glazed tiles’ mechanical performance and water resistance. By binding matrix particles and penetrating internal pores, the reinforcing agent decreases porosity and increases matrix density [43]. Consequently, mechanical strength increases and water absorption decreases. Si–O–Si linkages are formed when TEOS creates an inorganic network during hydrolysis and copolymerizes with the terminal hydroxyl groups of PDMS−OH. This strengthens the matrix even more by raising its Si–O–Si composition. Furthermore, the hydrophobicity of the matrix surface is significantly enhanced by the addition of organic groups from PDMS−OH and TEOS.

The effectiveness of the reinforcement treatment was evaluated based on several quantitative indicators, including mechanical strength improvement, surface integrity, and resistance to environmental degradation. Mechanical performance was assessed through compressive strength measurements, while microstructural integrity was examined using SEM observations. In addition, durability indicators such as water resistance were used to evaluate the long-term protective performance of the reinforcement materials.

## 7. Conclusion

The degradation mechanisms and conservation tactics for glazed tile components in historic structures in Shanxi Province were systematically analyzed and experimentally investigated in this work. The formation processes of common deterioration phenomena, such as glaze cracking, body powdering, and salt crystallization, were identified through field surveys of representative structures and laboratory simulations. These processes were mainly caused by environmental factors, such as temperature and humidity fluctuations, acid deposition, and moisture migration. Deteriorated ceramic bodies were consolidated using a hydroxyl-terminated PDMS−OH/TEOS reinforcement system with ethanol as the solvent based on these degradation patterns. An optimal formulation offering balanced protective performance was found after a thorough study that included color difference measures, surface water resistance tests, and mechanical property assessments. The findings show that this consolidation method successfully improves worn ceramic bodies’ mechanical integrity and surface stability while maintaining acceptable visual compatibility, which is essential for the preservation of architectural heritage materials.

Additionally, by optimizing the particle size distribution of the body raw materials and creating new glazed tiles, this study looked into a suitable replacement approach for glazed tile components that were seriously deteriorated and could not be structurally restored. The revised formulation improves the freshly manufactured tiles’ mechanical strength and structural density while maintaining morphological and material properties similar to those of conventional glazed ceramics, according to experimental results. This method provides a workable technical answer for replacing or repairing permanently damaged parts in historic structures. There are still a few restrictions, though. The intricate, long-term natural weathering that glazed tiles undergo in situ can only be partially replicated by simulated environmental trials. Extended aging studies are necessary to confirm the reinforcement materials’ long-term stability and endurance, including their resistance to UV radiation, cyclic wetness, and heat stress. Furthermore, more testing under actual exposure conditions is required to determine the long-term compatibility of newly manufactured tiles with original historical materials, taking into account physical, chemical, and aesthetic integration.

It should be emphasized that the accelerated aging results in this study are mainly used to compare degradation tendencies and reinforcement effects under controlled laboratory stresses. Because the combined actions of solar radiation, pollutants, biological colonization, salt crystallization, rainfall, seasonal humidity, and maintenance history in real ancient buildings are much more complex, extreme thermal cycling may generate damage mechanisms that are partly different from long-term natural weathering. Therefore, the reinforcement formulation proposed here should be regarded as a laboratory-validated conservation candidate rather than a complete prediction of in-situ service life. Long-term outdoor exposure tests and continuous monitoring on representative architectural components are necessary before large-scale conservation application.

In order to more fully evaluate the durability of reinforcement treatments, future research will focus on creating accelerated aging systems that more closely mimic natural environmental conditions by combining elements like UV radiation, temperature–humidity cycling, and air pollutants. The microstructural evolution and breakdown mechanisms of glazed tiles under environmental exposure will also be further investigated using sophisticated microanalytical methodologies and in situ monitoring techniques. Future research on conservation materials will also concentrate on creating new low-intervention inorganic–organic hybrid consolidation systems with better compatibility and stability, offering more efficient technical assistance for the long-term preservation of glazed architectural heritage.

## Supporting information

S1 File Raw experimental and survey data. This workbook contains the tabulated survey records and quantitative data used for the analysis of preservation status, water resistance, penetration depth, hardness improvement, and acid-rain degradation behavior of glazed tile components.
(XLSX)

## References

[1] Kaur M, Selvakumar P, Dinesh N, Chandel PS, Manjunath T. Reconstruction and preservation strategies for cultural heritage. Preserving cultural heritage in post-disaster urban renewal. IGI Global Scientific Publishing. 2025:385–412.

[2] Colomban P. Glazes and Enamels. Encyclopedia of Glass Science, Technology, History, and Culture. Wiley. 2021. 1309–25. 10.1002/9781118801017.ch10.6

[3] Wang H, et al. Study on the Glaze Technology and Surface Characteristics of Black Glazes from Yaozhou Kiln during the Tang, Song, Yuan, Ming, and Qing Dynasties. Journal of the European Ceramic Society. 2025;118007.

[4] Chen Y, Wang N, Yu L, Qian L, Zhou S. Analysis and Conservation of Glazed Decoration in Ancient Buildings in Shanxi, China. Coatings. 2025;16(1):14. doi: 10.3390/coatings16010014

[5] Li Q, Zhang F, Jia W, Guo Y. Study on the Surface Coating Techniques of Furniture in the Long’en Hall of Qing Changling Mausoleum. Coatings. 2025;15(6):712. doi: 10.3390/coatings15060712

[6] Ping M, et al. Application of smalt in building glazed tiles from Yuanmingyuan Park, Beijing in Qing dynasty. npj Heritage Science. 2025;13(1):282.

[7] Bai X, Zhang H, Tu Y, Sun S, Li Y, Ding H, et al. Preparation and Application of Apatite-TiO2 Composite Opacifier: Preventing Titanium Glaze Yellowing through Pre-Combination. Materials (Basel). 2024;17(5):1056. doi: 10.3390/ma17051056 38473529 PMC10934510

[8] Bulut HA, Şahin R. Radon, Concrete, Buildings and Human Health—A Review Study. Buildings. 2024;14(2):510. doi: 10.3390/buildings14020510

[9] Xia X, Liu J, Liu Y, Lei Z, Han Y, Zheng Z, et al. Preparation and Characterization of Biomimetic SiO2-TiO2-PDMS Composite Hydrophobic Coating with Self-Cleaning Properties for Wall Protection Applications. Coatings. 2023;13(2):224. doi: 10.3390/coatings13020224

[10] Shen J, Li L, Wang J-P, Li X, Zhang D, Ji J, et al. Architectural Glazed Tiles Used in Ancient Chinese Screen Walls (15th-18th Century AD): Ceramic Technology, Decay Process and Conservation. Materials (Basel). 2021;14(23):7146. doi: 10.3390/ma14237146 34885300 PMC8658199

[11] Zhao J, Luo H, Wang L, Li W, Zhou T, Rong B. TEOS/PDMS-OH hybrid material for the consolidation of damaged pottery. herit sci. 2013;1(1). doi: 10.1186/2050-7445-1-12

[12] Maravelaki P-N, Kapetanaki K, Stefanakis D. TEOS-PDMS-Calcium Oxalate Hydrophobic Nanocomposite for Protection and Stone Consolidation. Heritage. 2021;4(4):4068–75. doi: 10.3390/heritage4040224

[13] Li J, Fu S, Zhang T, Li J, Huo P, Li Y. Investigation of lightning damage mechanism and flashover channels on glazed roofing tiles of ancient buildings through laboratory experiments. Journal of Electrostatics. 2021;110:103553. doi: 10.1016/j.elstat.2021.103553

[14] Vasić MV, Mijatović N, Radojević Z. Aplitic Granite Waste as Raw Material for the Production of Outdoor Ceramic Floor Tiles. Materials (Basel). 2022;15(9):3145. doi: 10.3390/ma15093145 35591478 PMC9101581

[15] Wasserman R (Irina). Sustainable Ceramic–Adhesive Composites: Interfacial Degradation and Durability Under Environmental Stress. Buildings. 2026;16(4):751. doi: 10.3390/buildings16040751

[16] Mei D, Li L, Chen W, Cheng Y. Study on the Classification of Chinese Glazed Pagodas. Buildings. 2024;14(12):4084. doi: 10.3390/buildings14124084

[17] Sfez R, De-Botton S, Avnir D, Wakshlak R. Sol–gel glazes - a safe glass and ceramics coloring approach. J Sol-Gel Sci Technol. 2022;102(3):562–73. doi: 10.1007/s10971-021-05699-4

[18] Saito K, Toyoda H, Okada M, Oh J-S, Nakazawa K, Ban Y, et al. Fracture healing on non-union fracture model promoted by non-thermal atmospheric-pressure plasma. PLoS One. 2024;19(4):e0298086. doi: 10.1371/journal.pone.0298086 38626076 PMC11020618

[19] Kornreich A, Partridge D, Youngblood M, Parkins K. Rehabilitation outcomes of bird-building collision victims in the Northeastern United States. PLoS One. 2024;19(8):e0306362. doi: 10.1371/journal.pone.0306362 39110767 PMC11305546

[20] Uto M, Tsuruta J, Araki K, Ueno M. Item response theory model highlighting rating scale of a rubric and rater-rubric interaction in objective structured clinical examination. PLoS One. 2024;19(9):e0309887. doi: 10.1371/journal.pone.0309887 39240906 PMC11379165

[21] Pradell T, Molera J. Ceramic technology. How to characterise ceramic glazes. Archaeological and Anthropological Sciences. 2020;12(8):189.

[22] Ribeiro MJ, Tulyaganov D. Traditional ceramics manufacturing. Ceramics, Glass and Glass-Ceramics: From Early Manufacturing Steps towards Modern Frontiers. Springer. 2021:75–118.

[23] Youssef E, Mostafa N, Khoury J, Merhej T, Lteif R. Glaze surface defects causes and prevention controls. J Ceram Sci Technol. 2023;14(1):1–10.

[24] Mourou C, Zamorano M, Ruiz DP, Martín-Morales M. Characterization of ceramic tiles coated with recycled waste glass particles to be used for cool roof applications. Construction and Building Materials. 2023;398:132489.

[25] Balliana E, Caveri EMC, Falchi L, Zendri E. Tiles from Aosta: A Peculiar Glaze Roof Covering. Colorants. 2023;2(3):533–51. doi: 10.3390/colorants2030026

[26] Chen T, Gong B, Tang C. Origin and Evolution of Cracks in the Glaze Surface of a Ceramic during the Cooling Process. Materials (Basel). 2023;16(16):5508. doi: 10.3390/ma16165508 37629798 PMC10456388

[27] Vijerathne DT, Wahala SB, Asmone AS. Advancing environmental sustainability of ceramic tile production: a cradle-to-gate life cycle assessment case study from Sri Lanka. Frontiers in Built Environment. 2025;11:1654253.

[28] Hawryluk M, Marzec J. Problems related to the operation of machines and devices for the production of ceramic roof tiles with a special consideration of the durability of tools for band extrusion. Arch Civ Mech Eng. 2024;25(1). doi: 10.1007/s43452-024-01106-1

[29] Corregidor V, Ruvalcaba-Sil JL, Prudêncio MI, Dias MI, Alves LC. Ion Beam-Induced Luminescence (IBIL) for Studying Manufacturing Conditions in Ceramics: An Application to Ceramic Body Tiles. Materials (Basel). 2024;17(20):5075. doi: 10.3390/ma17205075 39459780 PMC11509279

[30] Feller RL. Accelerated aging: Photochemical and thermal aspects. Los Angeles, CA, USA: Getty Conservation Institute. 1994.

[31] Gritti N, Power RM, Graves A, Huisken J. Image restoration of degraded time-lapse microscopy data mediated by near-infrared imaging. Nat Methods. 2024;21(2):311–21. doi: 10.1038/s41592-023-02127-z 38177507 PMC10864180

[32] Ju S, Luo Q, Lu Z, Wang F, Shi J, Wang L, et al. A novel thermal-tailored strategy to mitigate thermal cracking of cement-based materials by carbon fibers and liquid-metal-based microencapsulated phase change materials. Construction and Building Materials. 2024;428:136338. doi: 10.1016/j.conbuildmat.2024.136338

[33] Es-Soufi H, Berdimurodov E, Sayyed MI, Bih L. Nanoceramic-based coatings for corrosion protection: a review on synthesis, mechanisms, and applications. Environ Sci Pollut Res Int. 2025;32(28):16978–7004. doi: 10.1007/s11356-023-31658-3 38183543

[34] Lin C, Li K, Li M, Dopphoopha B, Zheng J, Wang J, et al. Pushing Radiative Cooling Technology to Real Applications. Adv Mater. 2025;37(23):e2409738. doi: 10.1002/adma.202409738 39415410 PMC12160687

[35] Zhou B, Ma Q, Li Z, Zhang Z, Li N. Corrosion of glaze in the marine environment: study on the green-glazed pottery from the Southern Song “Nanhai I” shipwreck (1127–1279 A.D.). Herit Sci. 2023;11(1). doi: 10.1186/s40494-023-00965-w

[36] Song H, Chen Y, Zheng L. The Non-Destructive Testing of Architectural Heritage Surfaces via Machine Learning: A Case Study of Flat Tiles in the Jiangnan Region. Coatings. 2025;15(7):761. doi: 10.3390/coatings15070761

[37] Macedo MF, Vilarigues MG, Coutinho ML. Biodeterioration of Glass-Based Historical Building Materials: An Overview of the Heritage Literature from the 21st Century. Applied Sciences. 2021;11(20):9552. doi: 10.3390/app11209552

[38] Stanaszek-Tomal E. Microorganisms in Red Ceramic Building Materials—A Review. Coatings. 2024;14(8):985. doi: 10.3390/coatings14080985

[39] Oliwa K, Kozub B, Łoś K, Łoś P, Korniejenko K. Assessment of Durability and Degradation Resistance of Geopolymer Composites in Water Environments. Materials (Basel). 2025;18(16):3892. doi: 10.3390/ma18163892 40870210 PMC12387252

[40] Wu Z, Xu J, Ji Y, Fan H, Li L, Meng M. Tensile strength and deformation properties of fiber-reinforced loess: Laboratory and numerical investigation. Acta Geotech. 2025. doi: 10.1007/s11440-025-02769-7

[41] Abu Bakar NH, Wan Ismail WN, Mohd Yusop H, Mohd Zulkifli NF. Synthesis of a water-based TEOS–PDMS sol–gel coating for hydrophobic cotton and polyester fabrics. New J Chem. 2024;48(2):933–50. doi: 10.1039/d3nj03206j

[42] Sadeghi MA, Aghighi M, Barralet J, Gostick JT. Pore network modeling of reaction-diffusion in hierarchical porous particles: The effects of microstructure. Chemical Engineering Journal. 2017;330:1002–11. doi: 10.1016/j.cej.2017.07.139

[43] Njindam O, Njoya D, Mache J, Mouafon M, Messan A, Njopwouo D. Effect of glass powder on the technological properties and microstructure of clay mixture for porcelain stoneware tiles manufacture. Construction and Building Materials. 2018;170:512–9.

